# Evisceration of a Small Bowel Segment Through a Drain Site: Lesson Learnt

**DOI:** 10.7759/cureus.30996

**Published:** 2022-11-02

**Authors:** Nikolaos Papatheodorou, Dimitrios E Diamantidis, Sempachedin Perente, Sotirios Botaitis

**Affiliations:** 1 1st General Surgery Department, University Hospital of Alexandroupolis, Alexandroupolis, GRC

**Keywords:** drain site evisceration, complication, small bowel, drain, evisceration

## Abstract

Evisceration is described as the removal of intra-abdominal organs outside the abdominal cavity after partial or complete dehiscence of an operative incision. Multiple organs have been reported in the literature as being eviscerated through a drain site. Zero point five per cent (0.5) to 1.2% of all cases include the small bowel. In most cases, evisceration occurs three to eight hours post-operation. This article reports a case of an eviscerated small bowel segment through a drain site, along with the drain six hours post-operative. To our knowledge, such a complication following open abdominal or laparoscopic surgery has not yet been reported. Due to the imminent risk of strangulation and subsequent necrosis of the eviscerated visceral organ, drain site evisceration requires immediate intervention.

## Introduction

Intra-abdominal drainage systems are a common postoperative part of any emergency surgical operation. They prevent the collection of blood and infected fluids and the formation of an abscess, identify any anastomotic leakage, and contribute to the healing process [1-3]. Drain site complications are rare and include evisceration, herniation, haemorrhage, hollow viscus perforation, infection at the site, fistula formation, and kinking and knotting of drains [1,2,4,5]. Organs such as the omentum, gallbladder, appendix vermiformis, and small bowel have been reported that they can be eviscerated through a drain site [6-8]. Patients’ obesity and nutritional status, chronic use of corticosteroids, and frailty of elderly patients seem to contribute to such a complication. Moreover, the irregular shape of the draining tract, midline location of the drain site, long duration of surgery, peritonitis, increased intra-abdominal pressure, and the use of large diameter drain - tubes or flat drains, are also reported as risk factors [1,9-13]. This article reports a case of the evisceration of a small bowel segment through a corrugated drain site six hours after the patient underwent a subtotal cholecystectomy for gangrenous ischemic cholecystitis. In order to avoid occlusion immediately post-operative, the use of a corrugated drain type is deemed necessary. To our knowledge, this is the first case of small bowel evisceration through a drain site along with the drain.

## Case presentation

A 59-year-old man was diagnosed and hospitalized in the internal medicine department of the University Hospital of Alexandroupolis with uncomplicated acute cholecystitis. Past medical history was uneventful, apart from smoking, without former operations in the abdomen. Two days after his admission, he was transferred to our department with acute severe abdominal pain due to biliary peritonitis after perforation and rupture of the gallbladder confirmed by emergent ultrasound and computed tomography, which was grade II according to Tokyo guidelines. Imaging tests revealed free bile in the peritoneum cavity, and the patient was admitted to the operating room, where gangrenous ischemic cholecystitis was found. Subtotal cholecystectomy in a non-high-risk for surgery patient was deemed a safe solution and performed in order to avoid bile duct injury due to severe fibrosis and inflammation in Calot’s triangle. The operation was uneventful, and due to peritonitis, the placement of two surgical drains was considered crucial. A drainage tube was inserted and positioned in Morrison’s pouch to detect any early postoperative biliary leakage, whereas a flat drain was positioned in Douglas’s pouch.

**Figure 1:**
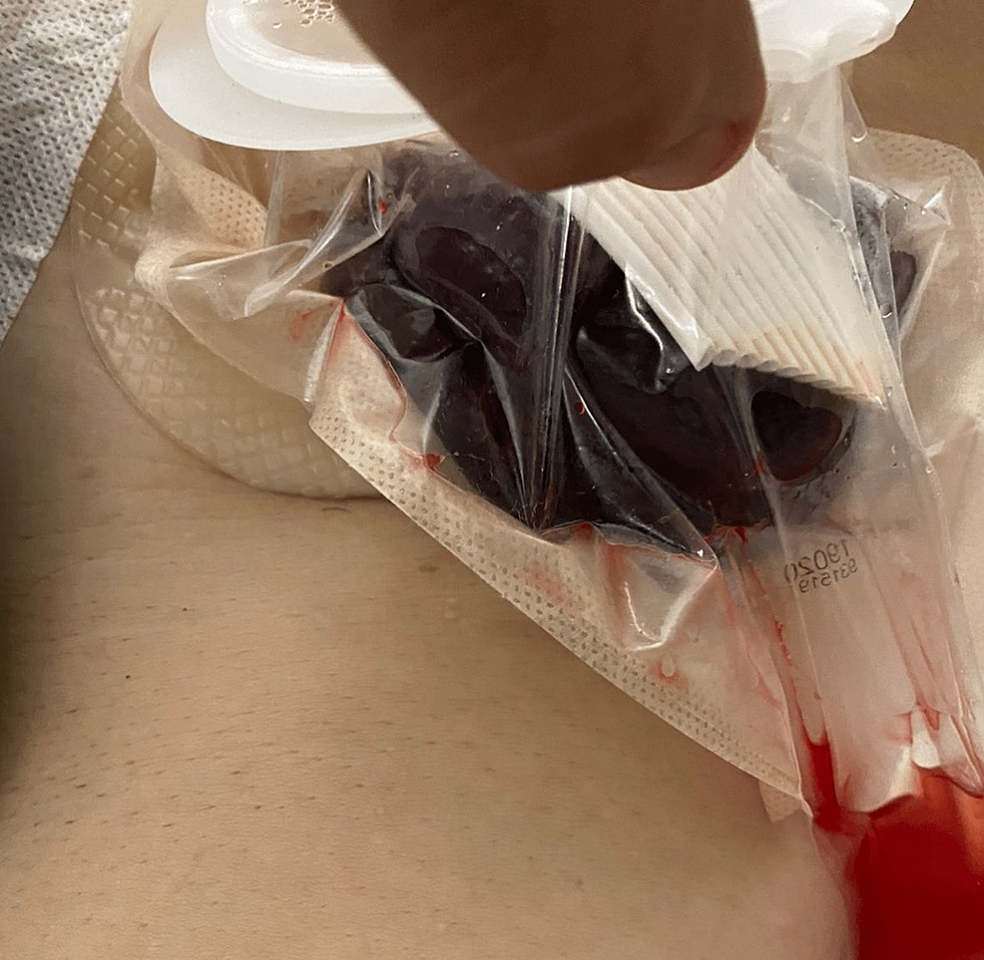
Evisceration of a small bowel segment through the flat drain site along with the drain

**Figure 2:**
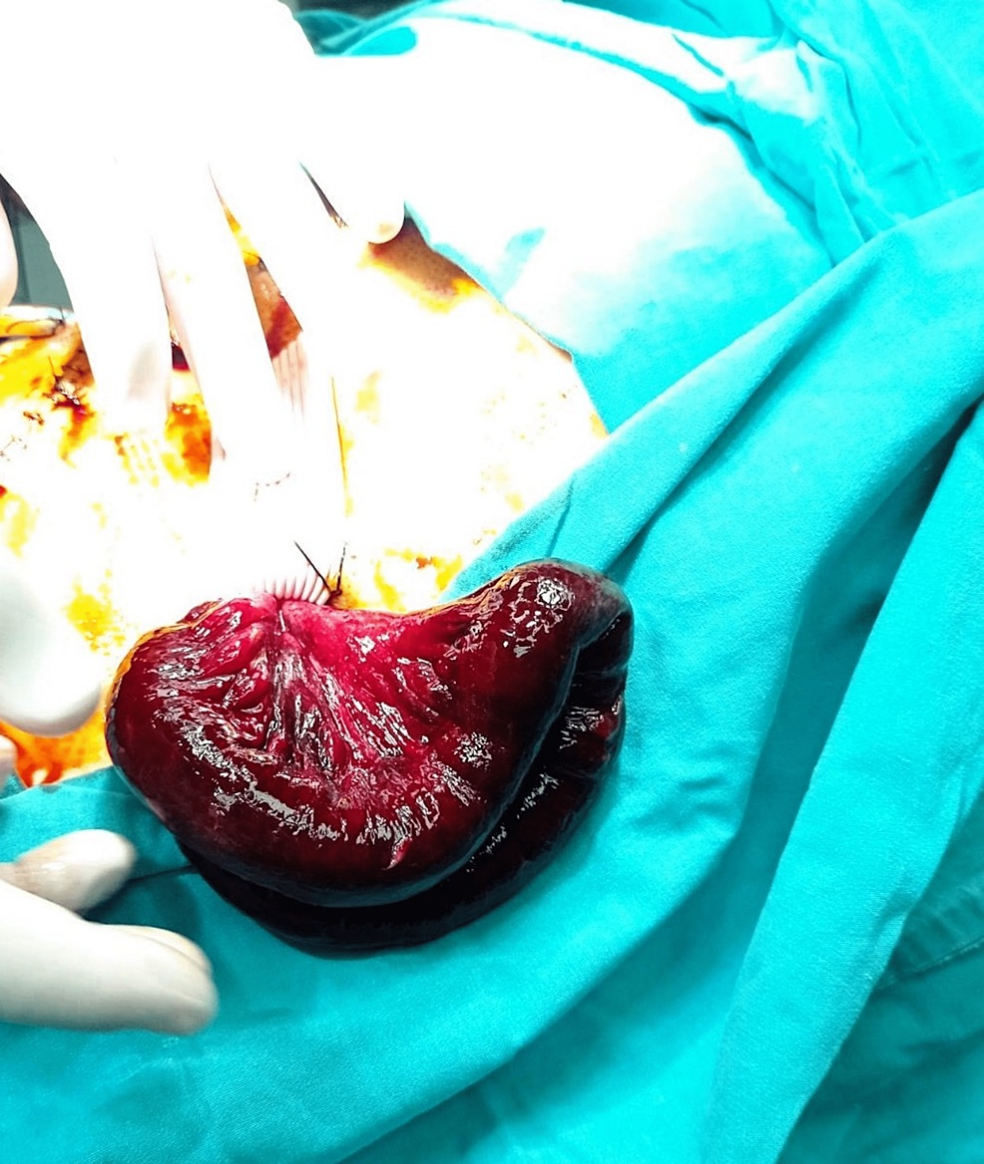
Evisceration of a small bowel segment through the flat drain site along with the drain, which cannot be reduced from the drain place

**Figure 3:**
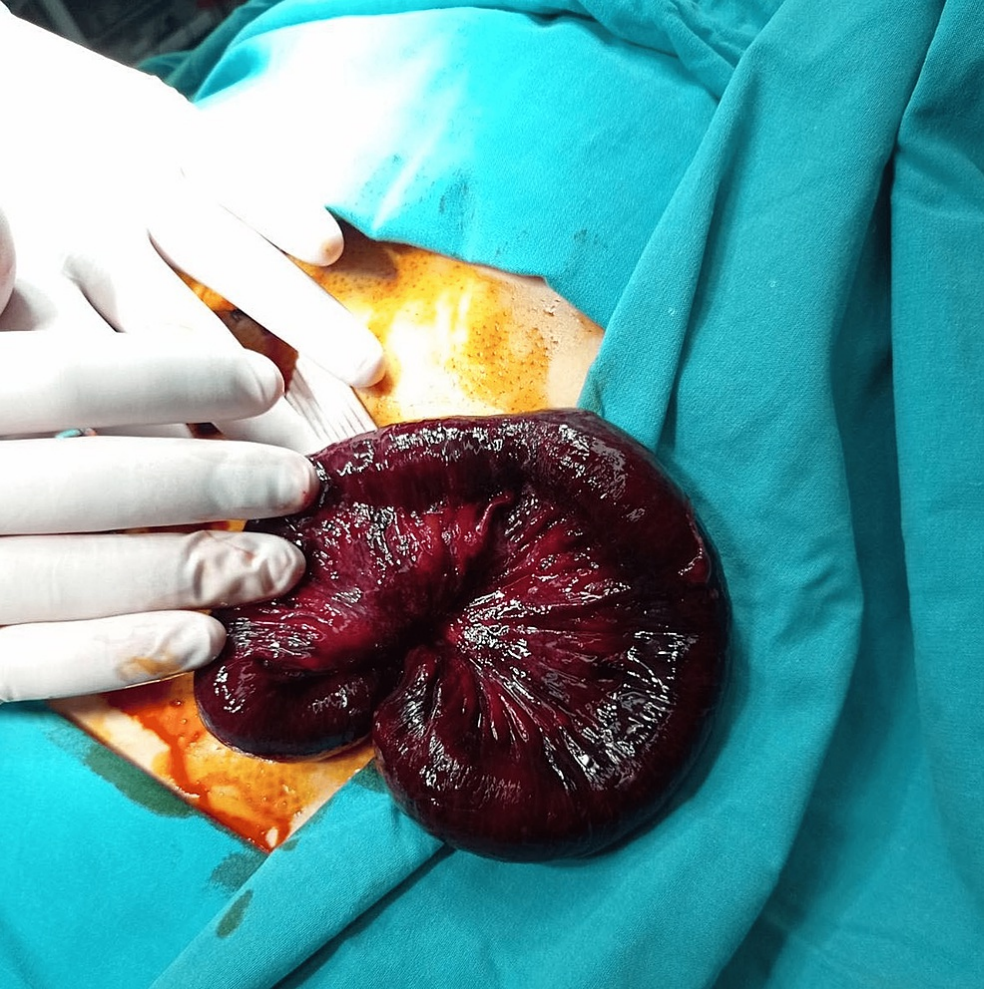
The view of the strangulated-eviscerated small bowel segment

**Figure 4:**
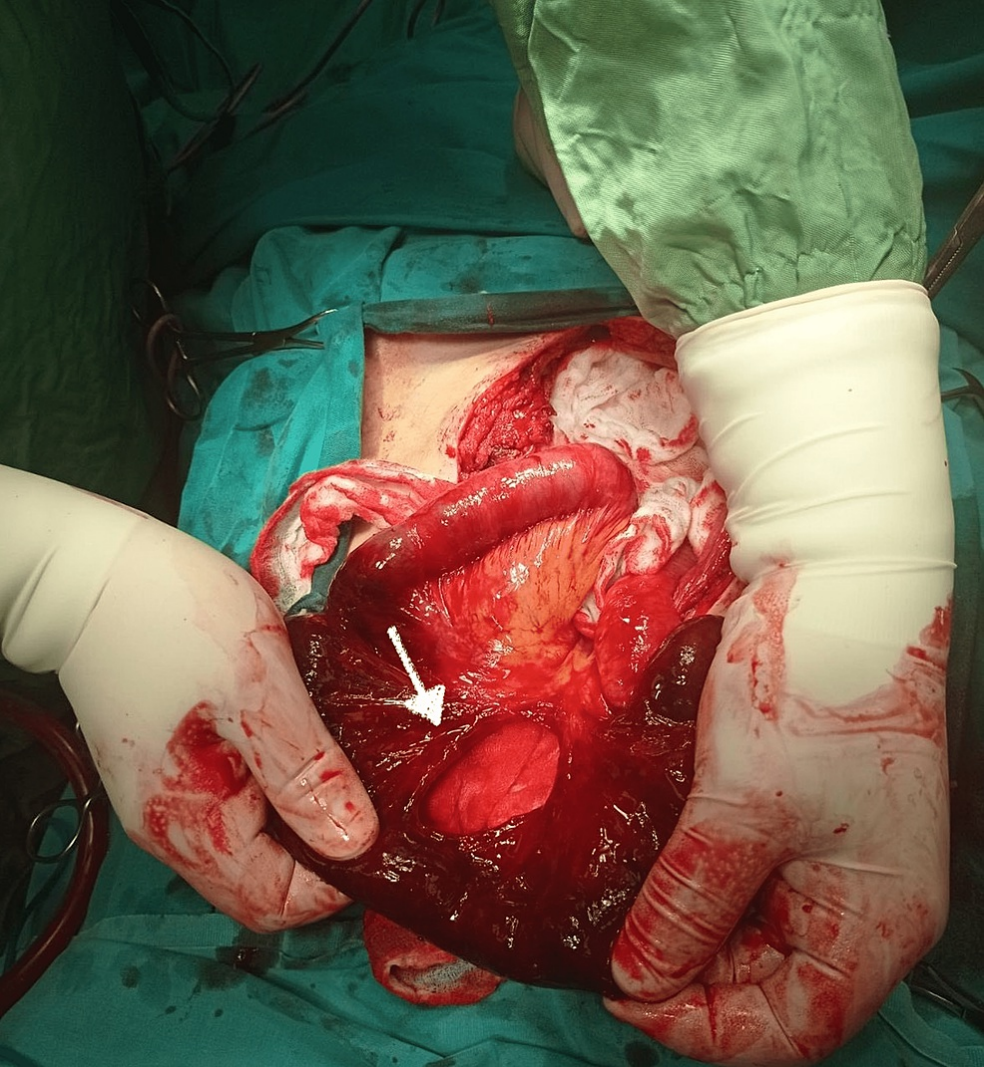
A limited mesenteric rupture (white arrow) was observed after the reduction of the ischemic small bowel segment back into the peritoneal cavity

**Figure 5:**
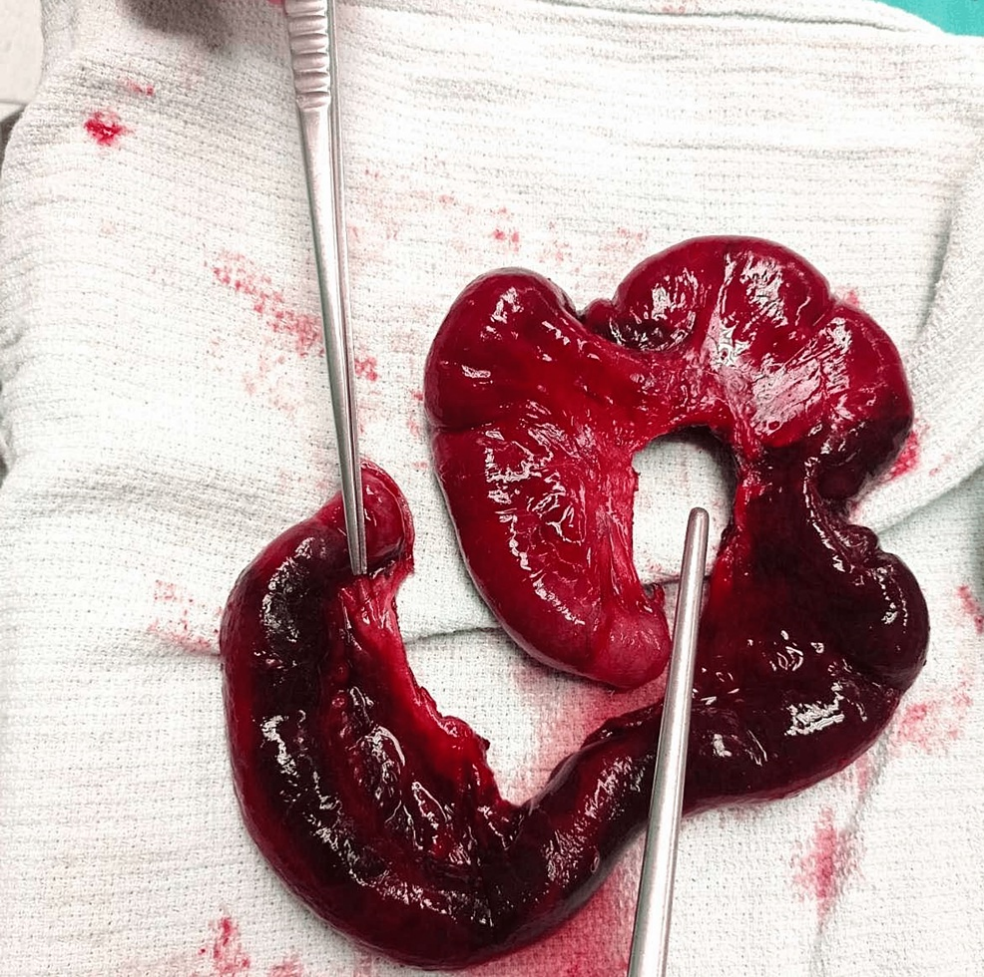
The ischemic bowel segment of about 30 cm in length was resected, as it was deemed non-viable

During his post-operative hospitalization, the patient suffered from a persistent cough and abdominal pain. Six hours after the operation, the patient developed an evisceration of a small bowel segment through the flat drain site along with the drain (Figure 1). Vital parameters were recorded (blood pressure: 110/70, pulse rate: 92 beats/min, serum lactate: 4.1 mmol/L). Bedside reduction was deemed unsafe due to the presence of ischemic changes on the surface of the small bowel (Figures 2, 3). Therefore, the patient was admitted to the operating room for an emergent laparotomy. Under general anaesthesia, we entered the peritoneal cavity through the old midline incision, and the segment of the incarcerated small bowel was reduced to the peritoneal cavity. A limited mesenteric rupture (white arrow) was observed, as well as an ischemic small bowel segment of about 30 cm in length was discovered at a distance of 1.5 m from the ileocecal valve (Figure 4). The ischemic bowel segment was resected, as it was deemed non-viable (Figure 5), and a side-to-side ileo-ileal anastomosis was performed. The flat surgical drain was left on site, and the drainage tube to Morrison’s pouch was changed to a flat drain, to prevent occlusion of the drain tube. The post-operative course was uneventful. Both drains were removed five days after the second operation, and the oral diet restarted on the seventh postoperative day. The patient was discharged home eight days after the second surgery. During the follow-up of one, three, and six months, we did not encounter any other complications.

## Discussion

Drain tube insertion remains a usual procedure in any emergency surgical operation. Abdominal drains are used to enhance the early detection of any gastrointestinal leakage and prevent the accumulation of biliary peritoneal, inflammatory fluid, or even blood [1]. Additionally, they contribute to preventing the formation of an abscess and to the healing process [2,3].

Complications from the placement of drainage tubes are not uncommon. Herniation, which may lead to small bowel obstruction [4,5], hollow viscus perforation during the placement of the drain tube, infection and drain site sepsis, fistulae formation, and haemorrhage [2], is the most common complication. Drain site evisceration, and kinking and knotting of drains, are quite rare, but they may necessitate an operation [2]. Therefore, the drain tube should be removed as soon as possible as it serves its purpose.

Multiple organs have been reported in the English literature as being eviscerated through a drain site. These were the omentum, gallbladder, appendix vermiformis, fallopian tube, and small bowel. In the aforementioned cases, evisceration occurred three to eight hours post-operation [2,6-8,11,14]. Drain site eviscerations along with the drain have not yet been reported as a complication following open abdominal or laparoscopic surgery. To our knowledge, this is the first case of small bowel evisceration through a drain site, along with the drain six hours post-operative.

Risk factors for evisceration through the drain site can be divided into patient and surgical technique factors. Patient factors include obesity, the frailty of elderly patients, the patient’s poor nutritional status, and chronic use of corticosteroids. Surgical factors are the irregular shape of a draining tract after multiple attempts at its creation, the midline location of a drain site, the long duration of a surgery/long, extended surgery time, and the use of drain tubes with a diameter >1 cm or flat drains. Moreover, peritonitis and increased intra-abdominal pressure also contribute to such a complication. Straining during defecation or micturition, cough, or vomiting, have been proposed as predisposing factors of increased intra-abdominal pressure [1,10-14]. In our patient, the predisposing factors were persistent post-operative cough that increased the intra- abdominal pressure, choleperitonitis, multiple attempts at the creation of the drain canal, and the use of a flat drain type.

Due to the imminent risk of strangulation and subsequent necrosis of the eviscerated organ, drain site evisceration requires immediate intervention. Mathew et al. proposed a bedside reduction of the strangulated, ischemic small bowel under local anaesthesia after extending the drain site incision and simultaneous resuscitation with warm gauze and 100% oxygen [2]. Spartalis et al. performed, on an urgent basis, a bedside appendectomy under sterile conditions, without anaesthesia or extending the drain site incision [15]. Some investigators support the bedside reduction of an eviscerated viscus with an extension of the drain site incision under local anaesthesia. In our case, this was deemed inappropriate due to the length (30 cm) of the necrotic-ischemic small bowel.

## Conclusions

In conclusion, to avoid such complications, surgeons should choose the appropriate type of drain according to the type of surgery and the patient’s condition. Additionally, intra-abdominals drains must be used if indicated, and their placement must be done carefully. Therefore, the dictum "when in doubt, drain" should be abandoned, and in emergency surgery, the dictum "no drainage at all is better than the ignorant employment of it" is more appropriate. Drain site evisceration requires immediate intervention. In case the eviscerated organ is deemed viable, bedside reduction should be performed; otherwise, laparotomy is the treatment of choice.
